# Clonally Focused Public and Private T Cells in Resected Brain Tissue From Surgeries to Treat Children With Intractable Seizures

**DOI:** 10.3389/fimmu.2021.664344

**Published:** 2021-04-06

**Authors:** Julia W. Chang, Samuel D. Reyes, Emmanuelle Faure-Kumar, Sandi K. Lam, Michael W. Lawlor, Richard J. Leventer, Sean M. Lew, Paul J. Lockhart, Kathryn Pope, Howard L. Weiner, Noriko Salamon, Harry V. Vinters, Gary W. Mathern, Aria Fallah, Geoffrey C. Owens

**Affiliations:** ^1^ Department of Neurosurgery, David Geffen School of Medicine at the University of California, Los Angeles, CA, United States; ^2^ Department of Medicine: Division of Digestive Diseases, David Geffen School of Medicine at the University of California, Los Angeles, CA, United States; ^3^ Department of Neurological Surgery, Feinberg School of Medicine, Northwestern University, Ann & Robert H. Lurie Children’s Hospital, Chicago, IL, United States; ^4^ Department of Pathology, Medical College of Wisconsin, Children’s Hospital of Wisconsin, Milwaukee, WI, United States; ^5^ Murdoch Children’s Research Institute, Royal Children’s Hospital, Parkville, VIC, Australia; ^6^ Department of Neurosurgery, Medical College of Wisconsin, Children’s Hospital of Wisconsin, Milwaukee, WI, United States; ^7^ Department of Pediatric Neurosurgery, Baylor College of Medicine, Texas Children’s Hospital, Houston, TX, United States; ^8^ Department of Radiological Sciences, David Geffen School of Medicine at the University of California, Los Angeles, CA, United States; ^9^ Department of Pathology and Laboratory Medicine, David Geffen School of Medicine at the University of California, Los Angeles, CA, United States; ^10^ Mattel Children’s Hospital, David Geffen School of Medicine at the University of California, Los Angeles, CA, United States

**Keywords:** T cell receptor, epilepsy, Rasmussen encephalitis, focal cortical dysplasia, tuberous sclerosis complex

## Abstract

Using a targeted transcriptomics approach, we have analyzed resected brain tissue from a cohort of 53 pediatric epilepsy surgery cases, and have found that there is a spectrum of involvement of both the innate and adaptive immune systems as evidenced by the differential expression of immune-specific genes in the affected brain tissue. The specimens with the highest expression of immune-specific genes were from two Rasmussen encephalitis cases, which is known to be a neuro-immunological disease, but also from tuberous sclerosis complex (TSC), focal cortical dysplasia, and hemimegalencephaly surgery cases. We obtained T cell receptor (TCR) Vβ chain sequence data from brain tissue and blood from patients with the highest levels of T cell transcripts. The clonality indices and the frequency of the top 50 Vβ clonotypes indicated that T cells in the brain were clonally restricted. The top 50 Vβ clonotypes comprised both public and private (patient specific) clonotypes, and the TCR Vβ chain third complementarity region (CDR3) of the most abundant public Vβ clonotype in each brain sample was strikingly similar to a CDR3 that recognizes an immunodominant epitope in either human cytomegalovirus or Epstein Barr virus, or influenza virus A. We found that the frequency of 14 of the top 50 brain Vβ clonotypes from a TSC surgery case had significantly increased in brain tissue removed to control recurrent seizures 11 months after the first surgery. Conversely, we found that the frequency in the blood of 18 of the top 50 brain clonotypes from a second TSC patient, who was seizure free, had significantly decreased 5 months after surgery indicating that T cell clones found in the brain had contracted in the periphery after removal of the brain area associated with seizure activity and inflammation. However, the frequency of a public and a private clonotype significantly increased in the brain after seizures recurred and the patient underwent a second surgery. Combined single cell gene expression and TCR sequencing of brain-infiltrating leukocytes from the second surgery showed that the two clones were CD8 effector T cells, indicating that they are likely to be pathologically relevant.

## Introduction

Involvement of the humoral and cellular effector arms of the adaptive immune system in seizures associated with infection and autoimmunity is well established (1–4). Less well studied is a potential role of the adaptive immune system in cases of drug resistant epilepsy in which a direct link to an infectious agent or underlying autoimmune disease has not been established. In the pediatric population many of the operable cases of intractable epilepsy are associated with developmental abnormalities in the cerebral cortex, which result from mutations that dysregulate the growth and differentiation of neuronal precursors causing focal cortical dysplasia (FCD), hemimegalencephaly (HME), and tuberous sclerosis complex (TSC) (5, 6). Aronica and colleagues first documented the presence of T cells in pathological specimens from FCD, and TSC surgery cases (7, 8). Higher numbers of CD8 T cells were reported in sections from FCD type II brain tissue compared with FCD I brain specimens; T cells were observed in close proximity to dysmorphic neurons and balloon cells (8), a distinguishing pathological feature of FCD type II compared with FCD type I (9). Likewise, CD8 T cells were found in TSC tubers surrounding dysplastic neurons and balloon cells (7). More recently Xu et al. reported that immune cells isolated from resected brain tissue from pediatric epilepsy surgeries comprised antigen experienced CD4, CD8 and Th17 γδ T cells (10). In agreement with these observations, we also found that effector memory T cells are present in brain tissue that has been removed to treat seizures associated with cortical malformations including FCD, TSC and HME (11). By mass cytometry approximately half of the CD8 αβ T cells that we isolated from FCD (n=4), TSC (n=2) and HME (n=1) surgery cases expressed CD69 (11), an activation marker (12). In the TSC and FCD cases, the αβ T cells isolated from the resected brain tissue comprised only a fraction of the brain-infiltrating leukocytes (BILs) ranging from 3.2% to 15.1%; the remaining cells were either γδ T cells, natural killer (NK) or myeloid cells (11). By contrast, almost half of the CD45^+^ immune cells isolated from the HME brain tissue comprised CD69^+^ CD4 αβ T cells (25.6%) and CD69^+^ CD8 αβ T cells (21.3%), suggesting an antigen driven T cell trafficking to the brain in this HME patient.

To investigate whether T cells in resected epileptogenic areas of the brain result from an adaptive immune response, we analyzed the T cell repertoires of a group of pediatric epilepsy surgery cases. Clonal restriction of T cells in the brain would be indicative of an antigen(s) driven immune response. We initially screened resected brain tissue from 53 pediatric epilepsy surgery cases for the expression of immune gene transcripts including five of the cases discussed above. We then sequenced T cell receptor (TCR) Vβ chains in brain tissue and blood from cases with the highest levels of T cell transcripts. We found that the T cell repertoires in the brains of these cases were skewed towards a limited number of private (patient specific) and public clones. Further, the third complementarity regions (CDR3) of many of the abundant public TCR sequences were strikingly similar to those that recognize immunodominant epitopes in human cytomegalovirus (HCMV), or Epstein Barr virus (EBV), or influenza virus A.

## Materials and Methods

### Patient Cohort

This study was approved by the UCLA Institutional Review Board (IRB no. 18-001048). All of the patients or their parents or legal guardians provided informed consent for the use of the surgical remnant and blood for research purposes according to the Declaration of Helsinki. There were no exclusion criteria, and no reported comorbidities. All specimens were collected using the same standard operating procedures. De-identified patient information including age at seizure onset, age at surgery, and gender was collected with informed consent.

### RNA Transcript Analysis

Bulk RNA was directly extracted from snap frozen blocks of involved brain tissue comprising grey and white matter from 53 pediatric epilepsy surgery cases (Direct-zol RNA miniprep kit; Zymo Research, Irvine, CA). A NanoString^®^ immune cell profiling oligonucleotide array (nCounter^®^ human immunology panel v2) was used to quantify transcripts expressed by T cells, macrophages and microglia (NanoString Technologies Inc, Seattle, WA). Data from five plates were combined and normalized and batch-corrected using the nSolver™ 4.0 analysis software according to the manufacturer’s protocol (NanoString Technologies Inc). For batch correction, RNA from the same surgery case was included in all five plates. The expression values were log2 transformed (**Table S3**), and subjected to principal components analysis (PCA) followed by hierarchical clustering of principal components using the R package FactoMinerR (13).

### T Cell Receptor Sequencing

Bulk genomic DNA was isolated from frozen blocks of fresh involved brain tissue from 13 of the 53 pediatric epilepsy surgery cases and whole blood from nine of these cases (Monarch^®^ genomic DNA purification kit, New England Biolabs, Ipswich, MA). Vβ chain TCR sequences were obtained using the ImmunoSEQ^®^ assay (Adaptive Biotechnologies, Seattle, WA). CDR3 sequences were manually curated to remove those that did not start with the third framework cysteine residue at position 104 (14). Clonality was calculated as described (15); comparison of proportions Chi-squared tests (16) were used to determine whether the frequency of the same Vβ CDR3 sequence was significantly different between brain and blood samples from the same patient. Whether Vβ CDR3 sequences corresponded to public T cell clones was determined by searching the immuneACCESS^®^ database (Adaptive Biotechnologies) and similarity to know anti-viral T cell clonotypes was determined using the VDJdb browser (17). Venn diagrams and heat maps were made with online tools (bioinformatics.psb.ugent.be/webtools/Venn, software.broadinstitute.org/morpheus/). Venn diagrams and heat maps were exported to CorelDraw2017 as scalable vector graphics and portable document format files respectively (Corel Corporation, Ottawa, Canada).

### Mass Cytometry

Peripheral blood mononuclear cells (PBMCs) were isolated from blood collected from Patients 595 and 597 at the time of surgery and from Patient 595 five months after surgery by density gradient centrifugation using Ficoll-Paque PLUS (GE Healthcare, Piscataway, NJ). Brain-infiltrating leukocytes (BILs) were isolated from a block of fresh involved 597 brain tissue by overnight digestion with Type IV collagenase (Worthington Biochemical Corp. Lakewood, NJ) followed by fractionation on a 70%:30% Percoll^®^ (Millipore Sigma, St. Louis MO) step gradient (18). All metal-tagged antibodies (Abs) were obtained from the Fluidigm Corporation (San Francisco, CA), and PBMCs and BILs were stained according to Fluidigm’s protocol as previously described (11). The antibody (Ab) panel comprised the following markers: CD45 (89Y or 115In), CD196 (141Pr), CD19 (142Nd), CD127 (143Nd), CD69 (144Nd), CD4 (145Nd), CD8a (146Nd), CD11c (147Sm), CD25 (149Sm), CD103 (151Eu), TCRγδ (152Sm), CD192 (153Eu), CD45RO (154Sm), CD279 (155Gd), CD183 (156Gd), CD33 (158Gd), CD197 (159Tb), CD14 (160Gd), CD152 (161Dy), CD27 (162Dy), CD56 (163Dy), CD38 (164Dy), CD16 (165Ho), CD28 (166Er), CD11b (167Er), CD206 (168Er), CD45RA (169Tm), CD3 (170Er), CD195 (171Yb), HLA-DR (174Yb), CD194 (175Lu), CD57 (176Yb). The Ab cocktail for staining PBMCs isolated from Patient 595 at the time of surgery (pre-surgery PBMCs) contained the CD45 Ab conjugated to Indium115 (115In) while the Ab cocktail for staining PBMCs from blood collected at a follow-up appointment after surgery (post-surgery PBMCs) contained the CD45 Ab tagged with Yttrium 89 (89Y). Likewise, the PBMCs and BILs from Patient 597 were stained with Ab cocktails containing CD45 Abs conjugated to 115In and 89Y respectively. To minimize technical variability, the 595 pre- and post- PBMCs were stained at the same time, then combined as a single sample and run on a Helios^®^ cytometry by time of flight (CyTOF) system (Fluidigm Corporation). The 597 BILs and PBMCs were also stained in parallel and then combined. Post-acquisition data normalization was done using bead-based normalization in the CyTOF software. Data were analyzed using Cytobank (19). Prior to analysis, events were gated to eliminate normalization beads, debris, dead cells and doublets. The 595 pre- and post- surgery PBMC data were then split into two separate FCS files by gating on the two different CD45 metal conjugated Abs. Likewise the 597 PBMC and BIL data were separated into two FCS files. To define subsets of immune cells the high dimensional data from 10,000 randomly selected cells from each sample were converted into a matrix of pair-wise similarities by implementing the t-based stochastic neighbor embedding (t-SNE) algorithm, followed by a density-based clustering method (ClusterX) (20).

### Immunocytochemistry

Adjacent sections (5 µm) of involved tissue were immuno-stained as previously described (21). Sections were stained with a CD3 mouse monoclonal Ab (clone F7.2.38, 1:500; Agilent Technologies Inc., Santa Clara, CA) and an Iba1 rabbit polyclonal Ab (1:500; Fujifilm Wako Chemicals Corp. Richmond, VA). Staining was visualized by adding 3, 3’-diaminobenzidine (Sigma-Aldrich, St. Louis, MO), followed by counterstaining with hematoxylin. Images were acquired using an Aperio ScanScope XT scanner (Aperio, Vista CA), then transferred to CorelPhoto-Paint, and rescaled from 72 dpi to 600 dpi. Images were then transferred to CorelDRAW2017; no changes were made to contrast or color balance.

### Single Cell RNAseq

BILs were isolated as described above from a block of fresh resected brain tissue from Patient 595. Two Chromium single cell gene expression libraries (5’ gene expression and TCR V(D)J) were prepared from the same cells (10X Genomics, Pleasanton, CA). Sequencing was performed on a NextSeq500 SBS sequencing v2 2x75 (Illumina Inc., San Diego, CA). Data were demultiplexed and aligned to GRCh38 transcriptome and VDJ references using Cell Ranger count and Cell Ranger VDJ pipelines (10X Genomics). Gene expression and TCR sequences in single cells were visualized with Loupe Browser and Loupe VDJ Browser respectively (10X Genomics). Single cell heat maps were exported to CorelDraw2017 as scalable vector graphics files.

## Results

### A Spectrum of Expression of Immune Genes in Resected Epileptogenic Brain Tissue

**Figure 1:**
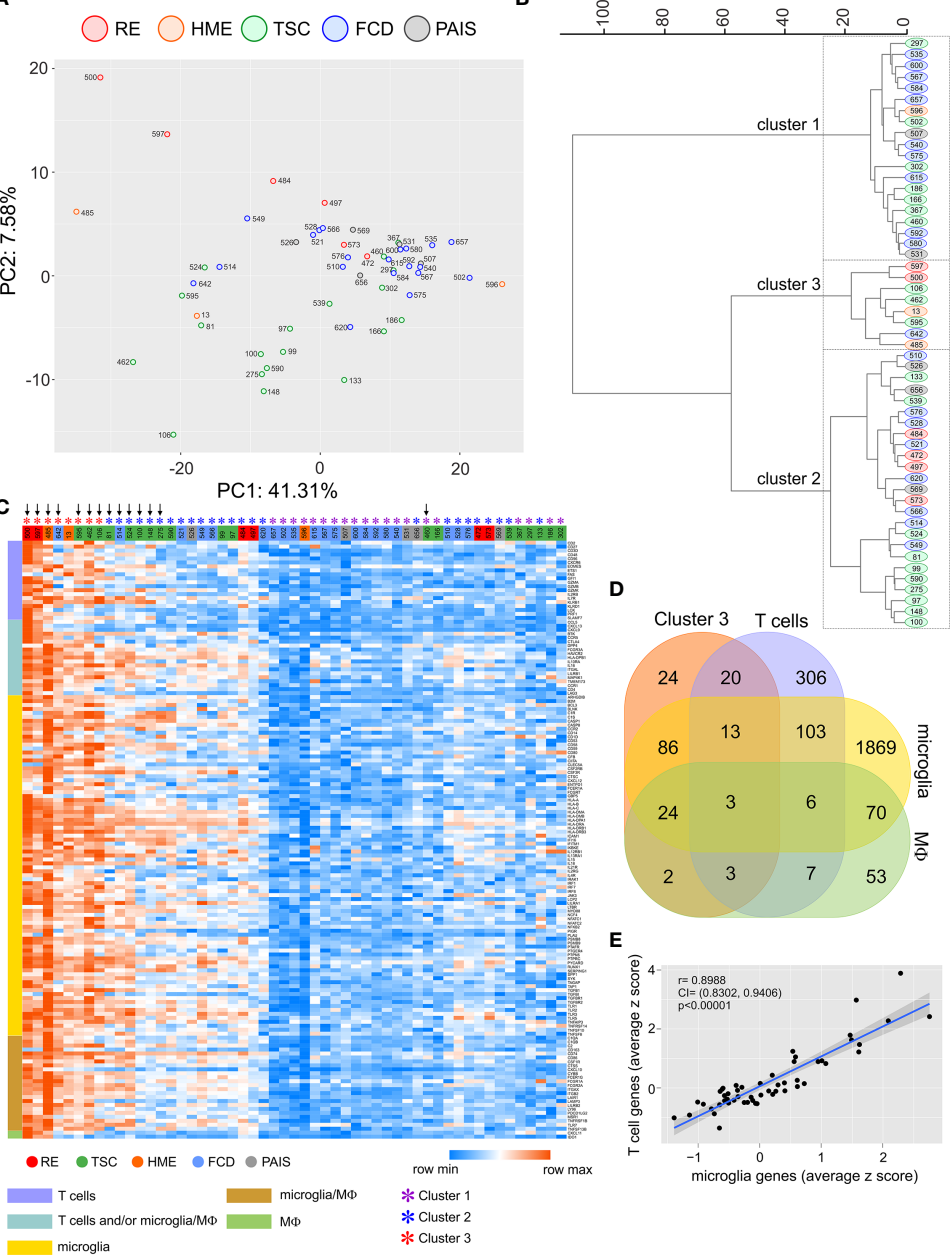
Quantification of immune cells transcripts in resected brain tissue from epilepsy surgeries. A NanoString^®^ immune cell profiling oligonucleotide array was used to quantify transcripts expressed by T cells, macrophages and microglia in brain specimens from 53 pediatric epilepsy surgery cases. **(A)** PCA plot calculated from the batch corrected normalized data. **(B)** Hierarchical clustering (Ward’s method) of the PCA coordinates resulted in three clusters. **(C)** Heat map after hierarchical clustering (Euclidean distances) showing the modified z scores (based on the median) of 86 percent of the genes whose expression was significantly higher (p < 0.0001) in Cluster 3 and could be assigned to T cells, microglia or macrophages (MФ). Vertical down arrows mark the cases from which TCR Vβ sequences were obtained. **(D)** Venn diagram showing the assignment of 151 out of 175 genes whose expression was significantly higher (p < 0.0001) in Cluster 3 to T cells, microglia or macrophages. **(E)** Plot showing a positive linear correlation between the expression of T cell specific genes and microglia specific genes in brain tissue from 53 epilepsy surgeries.

We used a NanoString^®^ immune cell profiling oligonucleotide array to measure the expression of immune genes in resected brain tissue from 53 pediatric patients presenting with intractable epilepsy who underwent surgery to control their seizures. Based on available clinical reports patients had been treated with at least one anti-epileptic drug; two of the RE patients (case IDs 484 and 573) had received intravenous injections of immunoglobulins. Patient 573 had also been treated with the corticosteroid prednisolone, as had TSC patient 595. From clinical and pathological evaluation, seizures were associated with TSC (n= 20), FCD (n= 20), RE (n= 5), perinatal arterial ischemic stroke (PAIS) (n= 5), and HME (n=3) (**Table S1**). PCA of the normalized batch expression of 577 genes (**Table S3**) followed by hierarchical clustering resolved the study cohort into three groups (**Figures 1A, B**). We selected 175 genes that defined Cluster 3 whose expression levels were significantly different from the other two clusters (p<0.0001), and assigned these genes to T cells, microglia and macrophages based on published single cell RNA sequencing data (**Figures 1C**, **D**) (22–25). Unsupervised re-clustering of the patient samples based on Euclidean distances calculated from the z scores of the 151 cell-assigned genes showed that the Cluster 3 brain specimens expressed the highest levels of T cell and microglia/macrophage transcripts. Specimens in Cluster 1 expressed the lowest levels of these immune cell transcripts, whereas the cases that comprised Cluster 2 split into two groups after re-clustering, a high/intermediate group and cases interspersed with those comprising Cluster 1 (**Figures 1B, C**). We found a highly significant correlation between the transcript levels of microglia specific and T cell specific genes likely reflecting the presence of higher numbers of both infiltrating T cells and microglia (**Figure 1E**). The brain tissue specimens with the highest expression of immune-specific genes were from two of the RE cases, which is known to be a neuro-immunological disease with a defining inflammatory component (26–28), but also from TSC, FCD, and HME surgery cases. Immunostaining adjacent sections of resected brain tissue from three TSC cases with a high immune gene expression signature showed CD3^+^ T cells and Iba1^+^ microglia in close proximity to dysmorphic cells (**Figure S1**). There was no significant correlation between the T cell gene expression signature and the patient’s age at the time of surgery or the time from seizure onset to surgery irrespective of gender (**Figure S2**).

### Clonally Focused T Cells Are Found in Resected Epileptogenic Brain Tissue

We isolated genomic DNA for Vβ chain TCR sequencing from 12 surgery cases with the highest T cell signatures based on the NanoString^®^ data. For eight of the cases, we also extracted genomic DNA from a sample of blood that was drawn just prior to the surgery. In addition, we extracted genomic DNA from brain and blood specimens from surgery case 460 because the same patient underwent a second surgery 11 months later (surgery case 524) (**Table S1**). As shown in **Figure 1C**, relatively fewer immune cell transcripts were found in brain tissue from the first surgery compared with the second surgery (460 versus 524).

**Figure 2:**
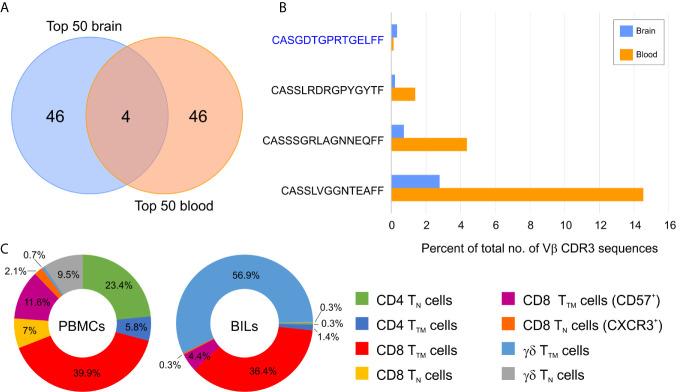
Overlap between abundant brain and blood T cell clonotypes in a Rasmussen encephalitis patient. **(A)** Venn diagram showing the overlap between the top 50 brain and top 50 blood T cell clonotypes in Patient 597. **(B)** Relative frequency of the four overlapping T cell clonotypes in blood and brain. The private Vβ CDR3 amino acid sequence is in blue, public Vβ CDR3 sequences are in black. **(C)** Donut plots showing the relative frequency of T cell subsets in CD45^+^ CD3^+^ cells in peripheral mononuclear cells (PBMCs) and brain-infiltrating leukocytes (BILs). T_N_, naïve T cells; T_TM,_ transitional memory T cells.

To assess how clonally restricted T cells were from the brain and the blood we calculated a clonality index, which is defined as one minus the normalized Shannon’s Diversity Index, and varies from 0, maximal diversity, to 1 in a completely oligoclonal sample, and can account for differences in sequencing depth between samples (15). As shown in **Table 1** the clonality indices in the brain varied, however they were significantly different overall compared with the blood (p= 0.01, Wilcoxon Signed-Rank for small samples). This implies that there must be some selective enrichment of T cell clones in the affected brain area of these patients. The clonality index for case 275 was very low suggesting that T cells in the affected area of the brain of this patient were not clonally restricted. Not unexpectedly the two RE cases (500 and 597) with the highest T cell gene expression signature (**Figure 1**) had the highest clonality scores in the brain (**Table 1**). The clonality score for T cells in blood from Patient 597, was also strikingly high, which is likely attributable to three public Vβ clonotypes that comprised 20.3% of the sample of the repertoire (**Figures 2A, B**). We surveyed the composition of PBMCs isolated from the same sample of blood and identified two populations of antigen experienced CD8 T cells that comprised ~50 percent of the T cells in the PBMCs, suggesting that the three dominant public clones may correspond to these T cells (**Figure 2C**). The same transitional memory T cells were also found in the brain of Patient 597 (**Figure 2C**). We ascribed a transitional effector to memory phenotype to these T cells, because they co-expressed CD45RO and CD45RA as well as HLA-DR, CCR5, PD1, CD28 and CD11b (**Figure S3**).

**Table 1:** Clonality indices.

Case ID	Brain	Blood
514	0.0100	0.0043
642	0.1057	0.0465
485	0.0638	0.0414
500	0.3702	0.0094
597	0.4281	0.2385
81	0.0742	n.a.
100	0.2546	n.a.
106	0.1480	n.a.
275	0.0087	n.a.
460	0.0340	0.04154
462	0.2173	0.0575
524	0.0811	0.0442
595	0.1084	0.0183

H = −Ʃ P_i_.log_2_P_i_ (P_i_ = freq. of a unique CDR3 amino acid sequence).

H_max_ = log_2_N (N = total no. of unique CDR3 amino acid sequences).

H_norm_ = H/H_max_.

Clonality index = 1-H_norm_.

n.a, not available.

### Public and Private TCR Vβ Clonotypes Are Found in Resected Epileptogenic Brain Tissue

**Figure 3:**
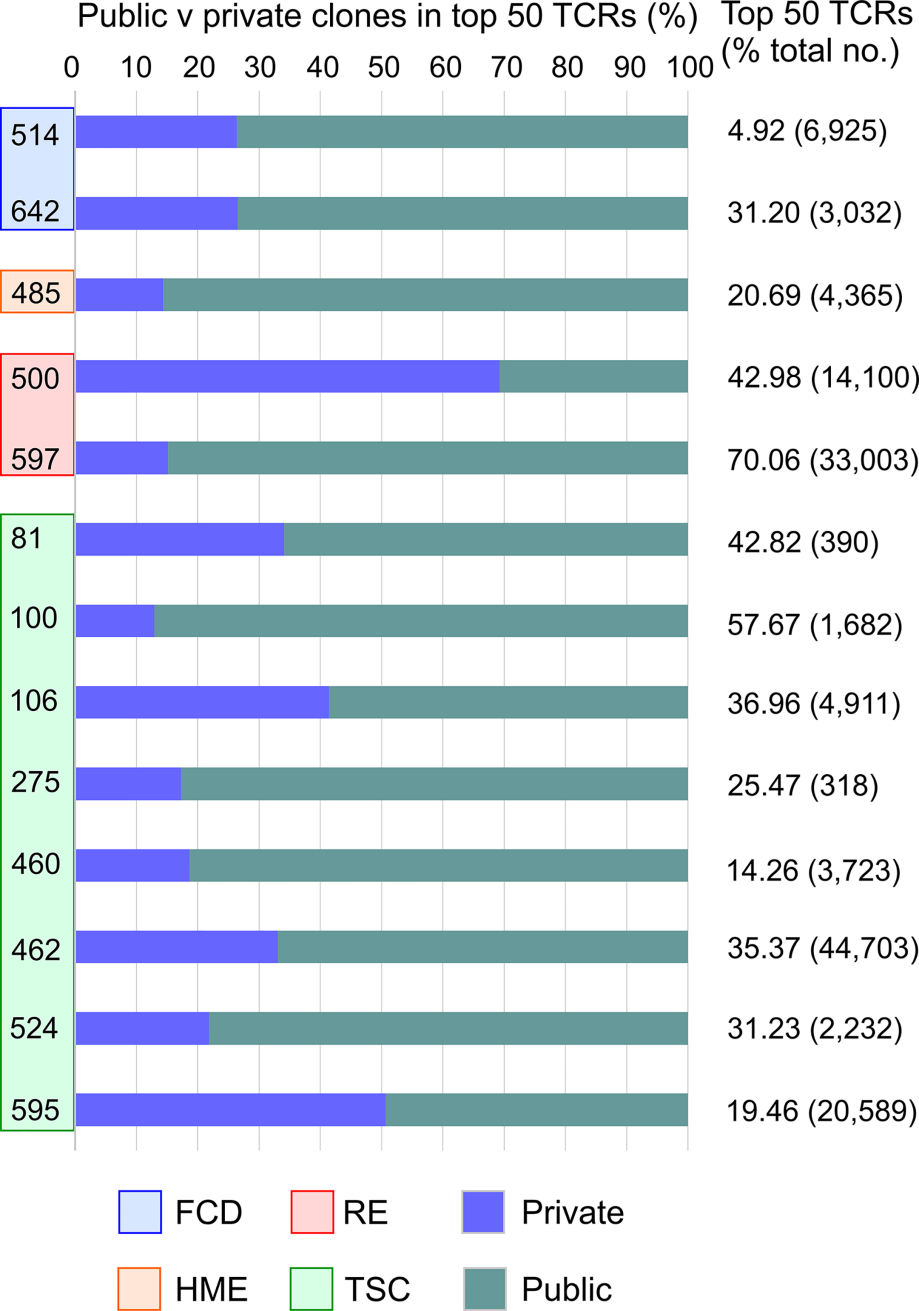
The top 50 T cell clonotypes in brain comprise public and private clonotypes. Bar graphs showing the relative proportion of public and private clonotypes in the top 50 T cell clonotypes from each patient along with the percentage of the total sampled repertoire that each of the top 50 clonotypes represents. The total number of clonotypes identified is shown in parentheses.

The top 50 Vβ CDR3 amino acid sequences in brain specimens from the 13 surgery cases accounted for 5-70 percent of the total number of Vβ CDR3 sequences that were sampled (**Figure 3**). They were made up of both private and public Vβ clonotypes based on a search of the immuneACCESS^®^ database (**Figure 3**). Comparison with TCR sequences in the iReceptor database (29) confirmed that the predominant private clones were patient-specific. In 11 out of the 13 surgery cases there was an even greater proportion of public than private Vβ clonotypes. In **Table 2** we list the Vβ CDR3 amino acid sequences from the most abundant public and private T cell Vβ clonotypes in each brain specimen. For patients where the same clonotype is present in the blood, we found that it is significantly enriched in the brain; we used a comparison of proportions Chi-squared test in order to take into account the different sample sizes. Because of the high proportion of abundant public Vβ clonotypes in the brain tissue, we asked whether they may be similar to TCRs that recognize common infectious agents. By searching the VDJ database (17), we found that the Vβ CDR3 amino acid sequence from the most abundant public Vβ clonotype in each surgery case is very similar to a sequence known to recognize an immunodominant viral epitope (**Table 2**). In the case of Patient 106 we found an exact match to a Vβ CDR3 sequence from a T cell that recognizes an Epstein Barr virus antigen presented by HLA-A*02, although we do not know whether Patient 106 has the HLA-A*02 allele. However, we determined that TSC patient 595 has the HLA-A*03 allele (**Table S2**), which has been shown to present the HCMV peptide KLGGALQAK to a TCR with a Vβ CDR3 sequence that differs by a single amino acid from the most abundant public Vβ CDR3 in the brain of this patient (**Table 2**) (17). The closest match to the TCR Vβ CDR3 sequence in the most abundant public Vβ clonotype in the brain of Patient 597 is a Vβ CDR3 sequence that recognizes the HCMV antigen KLGGALQAK restricted on HLA-A*03 (**Table 2**) (17). However, we found that this patient does not carry this allele (**Table S2**). There is a less stringent match to a Vβ CDR3 sequence (CSSPTRNTEAFF) that binds the HCMV pp65 peptide, TPRVTGGGAM bound to HLA B*07, which Patient 597 does carry. The most abundant public Vβ clonotype in the blood of this patient, which is also found among the top 50 Vβ clonotypes in the brain (**Figure 2**), may recognize another HCMV peptide bound by HLA A*01, a class I allele the patient also carries (**Table S2**). The Vβ CDR3 sequence, CASSLFGGNTEAFF, recognizes the pp50 peptide VTEHDTLLY and differs by a single amino acid from the Vβ CDR3 sequence found in the blood of Patient 597 (CASSLVGGNTEAFF, **Figure 2**).

**Table 2:** Frequency of the top private and public Vβ CDR3 sequences.

Case ID	Pathology	CDR3	percent in brain	percent in blood	p value	Similar virusspecific CDR3	Epitope	Antigen	Virus
514	FCD IIID	CASSLREGAQWVSPLHF	0.245	0.054	*p<0.0001*	none			
		CASATALNYGYTF	0.448	0.001	*p<0.0001*	CASASA-NYGYTF	NLVPMVATV	pp65	HCMV
642	FCD IIIB	CASSQLGTSKFNEQFF	3.496	0.285	*p<0.0001*	none			
		CASSSTVGGSTGELFF	4.914	0.065	*p<0.0001*	CASSS-VASGSTGELFF	KLGGALQAK	IE1	HCMV
485	HME	CASRAGTGGGQQPQHF	0.458	0.028	*p<0.0001*	none			
		CASSPGGFPLHF	2.543	1.655	*p<0.0001*	CASSPGG-LHF	KLGGALQAK	IE1	HCMV
500	RE	CASTHGHEEDSNQPQHF	10.255	0.007	*p<0.0001*	none			
		CASSYRQAGEAFF	1.695	0.011	*p<0.0001*	CASSLRQGEAFF	NLVPMVATV	pp65	HCMV
597	RE	CASTTEPGTPNTGELFF	1.873	0.006	*p<0.0001*	none			
		CASSFFTNTEAFF	24.437	0.015	*p<0.0001*	CASSFFGNTEAFF	KLGGALQAK	IE1	HCMV
81	TSC	CASSEWSGRASLDTQYF	1.795	n.a.	n.d.	none			
		CASSTTGMGSPLHF	11.795	n.a.	n.d.	CASSPTGGGSPLHF	GILGFVFTL	M	Influenza A
100	TSC	CSALDGNRRRNQPQHF	1.308	n.a.	n.d.	none			
		CASSRGSVAPGELFF	20.630	n.a.	n.d.	CASSKGSVAPGELFF	GLCTLVAML	BMLF1	EBV
106	TSC	CASSQELTGLAGYTF	8.797	n.a.	n.d.	none			
		CASSKQGSTEAFF	4.134	n.a.	n.d.	CASSKQGSTEAFF	GLCTLVAML	BMLF1	EBV
275	TSC	CASSEIVGQGDHF	0.629	n.a.	n.d.	none			
		CASSFGTTEQYF	0.943	n.a.	n.d.	CASSFGGT-QYF	GILGFVFTL	M	Influenza A
460	TSC	CASSPGTGGLFFSSGELF	0.645	0.0	n.d.	none			
		CASSDPWYEQF	1.424	0.003	*p<0.0001*	CASSDS-YEQYF	KLGGALQAK	IE1	HCMV
462	TSC	CSARDYRNPKVTEAFF	1.673	0.344	*p<0.0001*	none			
		CASSQDGQGDQPQHF	6.201	0.027	*p<0.0001*	CASSQ-GQGDQPQHF	KLGGALQAK	IE1	HCMV
524	TSC	CALLRPGPSYEQYF	2.151	0.003	*p<0.0001*	none			
		CASSLIDGYNEQFF	2.912	0.005	*p<0.0001*	CASSLISGYNEQFF	KLGGALQAK	IE1	HCMV
595	TSC	CATSDLDGREAGELFF	2.442	0.001	*p<0.0001*	none			
		CASSPLVDNYGYTF	0.471	0.001	*p<0.0001*	CASSGLVDNGYTF	KLGGALQAK	IE1	HCMV

Private clones are in blue, public clones are in black; p values are calculated by applying a Chi-squared proportions test to the frequency of a Vβ CDR3 sequence in the brain versus the blood; amino acids in red denote substitutions, inserted amino acids are underlined, and hyphens denote a deleted residue in the virus-specific Vβ CDR3 sequences compared with most abundant Vβ CDR3 sequences in the brain; n.a, not available; n.d, not determined.

### Changes in the TCR Repertoire Between the First and Second Surgery

As previously mentioned, surgery cases 460 and 524 correspond to the same TSC patient who was operated on twice within 11 months to remove epileptogenic tubers. The most abundant public and private TCR Vβ clonotypes in the brain from the two surgeries are not the same (**Table 2**). We measured the overlap between the sampled brain and blood repertoires from the two surgeries, and found 59 Vβ CDR3 sequences that are common to the four samples (**Figure S4**). We focused on the sequences whose frequency differed significantly between either blood from the first and second surgeries or between brain tissue from the first and second surgeries. As shown in **Table 3**, the frequencies of 14 public and private Vβ clonotypes were significantly higher in brain tissue from the second surgery compared with brain tissue from the first surgery. The frequency of only one Vβ clonotype significantly increased in both the blood and the brain; this clone was among the top 50 in each sample (**Table 3**).

**Table 3:** Changes in the frequencies of T cell clonotypes in blood and brain specimens from the first and second surgeries.

Vβ CDR3	Blood		Brain		Top 50 Blood		Top 50 Brain	
	460→524	p value	460→524	p value	460	524	460	524
CASSSRRINQPQHF	↑	0.0127	↑	<0.0001	✓	✓	✓	✓
CASSKGVRAMSGNTIYF	↑	0.032	n.c.	0.2967				
CASSWGLTGGVSEQFF	↑	0.0352	n.c.	0.4138	✓	✓	✓	✓
CASSVGRLAGGTYEQYF	↑	0.0423	n.c.	0.1437				
CASSVNYSNQPQHF	↑	0.0001	n.c.	0.5407	✓	✓	✓	✓
CACPDRGSGNTIYF	↑	0.0002	n.c.	0.7153		✓		
CASSLVAGARGYTF	↑	0.032	n.c.	0.7153				
CASSTWGRTYEQYF	n.c.	0.5196	↑	0.0496				
CASSHSGGNYEQYF	n.c.	0.7521	↑	0.0001			✓	✓
CASSLDSSGGANNEQFF	n.c.	0.7945	↑	0.0014	✓	✓		✓
CASSWTGLGAYEQYF	n.c.	0.9181	↑	0.0207	✓	✓	✓	✓
CASSEWLNQPQHF	n.c.	0.9314	↑	0.0006	✓	✓		✓
CASSLGPGGSYEQYF	n.c.	0.4426	↑	<0.0001	✓	✓	✓	✓
CASSSGGAPANEKLFF	n.c.	0.6341	↑	<0.0001				✓
CASSWTYKVNEQFF	n.c.	0.9181	↑	0.0157	✓			✓
CASRWFSVYEQYF	n.c.	0.2231	↑	0.0185	✓	✓	✓	✓
CASSLKTFSSGEQYF	n.c.	0.7981	↑	0.0203				✓
CASSINGASHHTNEKLFF	n.c.	0.9761	↑	0.0496				
CASRPSGLSGEQYF	↓	0.0018	↑	<0.0001	✓	✓	✓	✓
CASSLSGGNYEQYF	↓	0.0066	↑	0.0006	✓		✓	✓
CASSLGDNYGYTF	↓	0.0005	n.c.	0.7153	✓	✓		
CASSLDGGGSGYEQYF	↓	0.0276	n.c.	0.7153	✓	✓		
CASRKQGPRVEQYF	↓	0.0004	n.c.	0.2967	✓	✓		

Private clonotypes are in blue; public clonotypes are in black; ↑ increase in frequency; ↓ decrease in frequency.

✓ present in the top 50 blood or brain clonotypes; n.c, no change.

### Changes in the TCR Repertoire in the Blood After Epilepsy Surgery

**Figure 4:**
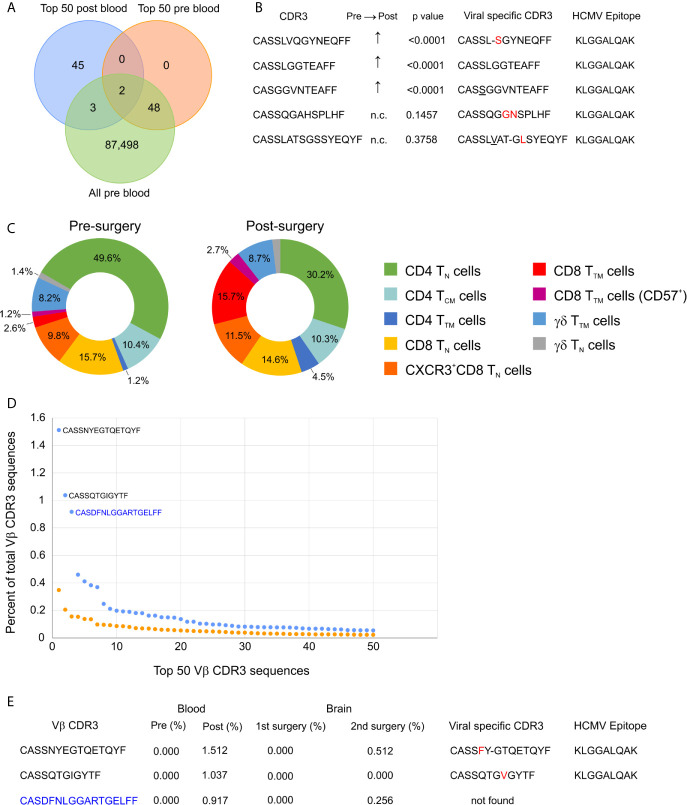
Longitudinal profiling of T cells in a TSC patient. **(A)** Venn diagram showing the overlap between the top 50 clonotypes present in the blood 5 months after surgery (post blood) and clonotypes present in the blood just prior to the surgery (pre blood). **(B)** Vβ CDR3 sequences of the five overlapping T cell clonotypes; the frequency of the three that were not among the top 50 clonotypes in the pre blood are significantly higher in the post blood. Comparison of the five Vβ CDR3 sequences to CDR3 sequences that recognize the same HCMV epitope restricted on HLA A*03. Amino acids in red are substitutions and underlined residues are additions; a dashed line indicates a deletion. **(C)** Donut plots showing the relative frequency of T cell subtypes in CD45^+^ CD3^+^ cells from pre- and post-surgery blood. T_N_, naïve T cells; T_TM,_ transitional memory T cells; T_CM_, central memory T cells. **(D)** Comparison of the frequencies of the top 50 clonotypes in pre- and post- surgery blood showing that there are three dominant clonotypes in the post blood reflecting an increase in the clonality score from 0.018 (**Table 1**) to 0.075. The two public Vβ CDR3 sequences are in black and the private Vβ CDR3 sequence is in blue. **(E)** The three dominant clonotypes in the post-surgery blood were not found in the blood or brain from the first surgery, but were found in the single cell TCR sequences from the second surgery. The two public Vβ CDR3 sequences are similar to virus specific Vβ CDR3 sequences restricted on HLA-A*03. Amino acids in red are substitutions and a dashed line indicates a deletion.

We collected blood from Patient 595 during a clinic visit five months after the surgery; at this time the patient was reported to be seizure free although still taking anti-seizure medications. We obtained TCR Vβ CDR3 sequences from genomic DNA, and compared them to the TCR Vβ CDR3 sequences in blood collected at the time of the surgery and to the top 50 TCR Vβ CDR3 sequences in brain tissue from the surgery. Thirty-four of the top 50 brain Vβ clonotypes were present in both blood samples, and the frequency of 18 of these Vβ clonotypes had significantly decreased in the blood after the surgery, while the remaining 16 had not changed (**Table 4**; **Figure S5**). This suggests that removal of the source of seizure activity may have led to the contraction of specific T cell clones. However, the overall clonality index of the TCR repertoire in the blood had increased from 0.0183 to 0.0747 suggesting that there were other clones that may have expanded. Comparing the top 50 Vβ CDR3 sequences from the post-surgery blood draw with the sampled repertoire from the pre-surgery blood draw revealed five overlapping clones three of which had significantly expanded; the relative frequency of the remaining two clones that were also among the top 50 clones from the pre-surgery blood draw had not changed (**Figures 4A, B**). The Vβ CDR3 amino acid sequences of four of the Vβ clonotypes were very similar to Vβ CDR3 sequences that recognize the same epitope from HCMV restricted on HLA A*03; the sequence of the fifth Vβ clonotype was a perfect match (**Figure 4B**). As previously mentioned, we had determined that Patient 595 carries the HLA A*03 allele (**Table S2**). We characterized the PBMCs in pre- and post-surgery blood by mass cytometry and found that there was an approximately five-fold increase in CD8 transitional memory T cells (**Figure 4C**; **Figure S6**). In addition to the expanded Vβ clonotypes we found that the three most abundant Vβ clonotypes in the sample of post-surgery blood were not found in the pre-surgery blood nor the brain (**Figures 4D, E**). The depth of coverage of the post-surgery blood was higher than the pre-surgery blood (87,551 versus 372,380 different TCR Vβ CDR3 amino acid sequences), thus we cannot exclude the possibility that the sampling of the TCR repertoire in the pre-surgery blood was not sufficient to capture these clones.

**Table 4:** Decrease in frequency of the top 50 brain clonotypes in post-surgery blood.

Vβ CDR3	pre → post	p value
CAMDKGEAFF	↓	<0.0001
CASRVIYGYTF	↓	<0.0001
CASSLREARWNTQYF	↓	<0.0001
CASSPRTANNEQFF	↓	<0.0001
CASSSTKREAVQETQYF	↓	<0.0001
CSAWTGSMNTEAFF	↓	<0.0001
CASSLGAGHGYTF	↓	0.0001
CASSPLNNEQFF	↓	0.0012
CASSLSGGFTGELFF	↓	0.0024
CASSLGDYGYTF	↓	0.0044
CALREVDGYTF	↓	0.0045
CASDPGELFF	↓	0.0045
CASSPAGVSPLHF	↓	0.0076
CAIRDGQGGYEQYF	↓	0.0079
CAILKSLDGYTF	↓	0.015
CASLTITGQGFYEQYF	↓	0.0165
CASSLAGRLAF	↓	0.0228
CASSLAPSGYNEQFF	↓	0.0379

Private clonotypes are in blue; public clonotypes are in black; ↓ decrease in frequency.

### Public and Private CD8^+^ T Cell Clones With an Effector Phenotype in Epileptogenic Brain

**Figure 5:**
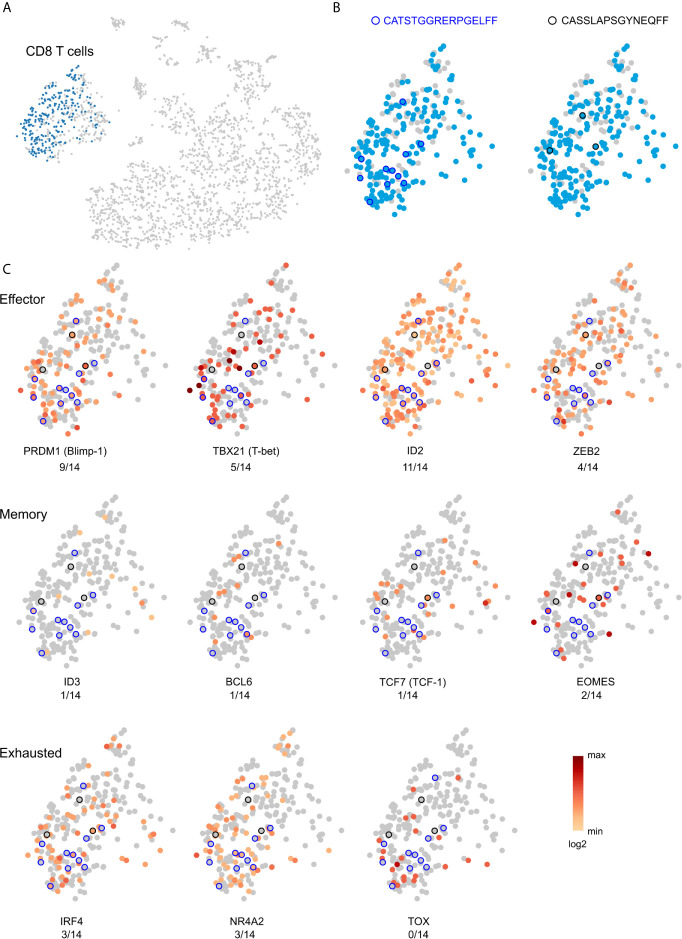
Effector phenotype of public and private TCR clonotypes. **(A)** t-SNE plot, based on single cell RNA expression data, of brain-infiltrating leukocytes from Patient 595’s second surgery. CD8 T cells are colored blue. **(B)** Location within the cluster of T cells of the two clonotypes that overlap between the post-surgery blood and brain (**Figure 4**). Clonotypes are circled in black. **(C)** Single cell heat maps for transcription factors that define effector, memory, and exhausted CD8 T cells. Of the 11 T cells that express the two TCRs, the majority of the cells likely correspond to effector T cells.

A month after we had collected the blood sample, Patient 595 developed seizures, which progressively worsened leading to a second surgery six months later to remove another tuber from a different area of the brain. We received a portion of this tuberous tissue, and isolated a fresh leukocyte fraction for single cell RNA sequencing. Bar-coded libraries were made to enumerate gene transcripts and determine TCR sequences. Although we expected to obtain fewer TCR sequences (30), we chose this approach in order to determine the phenotype of the most frequently expressed TCR Vβ clonotypes, and to obtain both TCR Vα and Vβ sequences for future synthesis and cloning of the receptors. We obtained RNA transcript data from 2,694 cells of which 391 were T cells (**Table S4**). To compare with our existing TCR sequence data from this patient we only considered barcodes assigned to one unique Vβ chain sequence, which corresponded to 382 T cells expressing 323 different Vβ chain sequences (**Table S4**). More than one Vβ was assigned to seven barcodes, and only a Vα chain was assigned to two barcodes (**Table S4**). As shown in **Figure 4E**, two of the most abundant Vβ clonotypes found in the blood six months before the second surgery were among the T cells isolated from the resected tuber. We also found that 14 of the top 50 Vβ clonotypes in brain tissue from the first surgery were also among the T cells isolated from the second resected tuber (**Figure S7**; **Table 5**). The frequencies of two of the overlapping Vβ clonotypes were significantly higher in the sampled repertoire from the second surgery, while a third Vβ clonotype had decreased; frequencies of the remaining Vβ clonotypes had not changed significantly (**Table 5**). The two Vβ clonotypes that had increased were the two most abundant comprising ~4.5% of the T cells (**Table S4**). We combined the TCR and transcript data to determine the phenotype of these two clones, and it appears, based on transcription factor expression that they are primarily CD8 effector T cells (**Figure 5**). This would imply that at the time of the second surgery there was an active immune response occurring in the brain of this patient.

**Table 5:** Changes in the frequency of T cell clonotypes in brain tissue from the first and second resective surgeries performed on TSC patient 595.

CDR3	1^st^ → 2^nd^ surgery	p value
CATSTGGRERPGELFF	↑	p<0.0001
CASSLAPSGYNEQFF	↑	p<0.0001
CSAPVRLALFYEQYF	↓	0.021
CASSPAGVSPLHF	n.c.	0.386
CAMDKGEAFF	n.c.	0.972
CASSPALAGGFTGELFF	n.c.	0.559
CASSPLNNEQFF	n.c.	0.726
CALREVDGYTF	n.c.	0.785
CASTLAGVATF	n.c.	0.785
CASSLILIYGYTF	n.c.	0.799
CASSPLVDNYGYTF	n.c.	0.908
CASSLGDYGYTF	n.c.	0.609
CALGGLGELFF	n.c.	0.811
CASSVAGYGYTF	n.c.	0.966

Private clonotypes are in blue; public clonotypes are in black;

↑ increase in frequency; ↓ decrease in frequency; n.c. no change.

## Discussion

We have quantified immune cell transcripts in brain tissue that was removed to treat children with medically refractory seizures, and assigned genes to T cells and microglia/macrophages based on published single cell RNAseq data. Unsupervised clustering of the data revealed a spectrum of involvement of innate and adaptive immune cells with a subset of patients having a higher inflammatory signature. Two RE cases led this high inflammation group, which also included TSC, FCD, HME and PAIS cases. The other four RE cases in the study cohort clustered with TSC and FCD cases that had lower z scores for immune gene expression, likely reflecting fewer T cells and microglia in the resected tissue that was analyzed. This suggests that the extent of T cell involvement is variable between patients with the same diagnosis even in the case of RE. This variability did not appear to be related to the gender or age of the patient or seizure duration, at least in our study cohort.

We obtained TCR Vβ sequencing from 12 patients with the highest T cell gene expression signature. From the clonality indices and the frequency of the top 50 we conclude that T cells are clonally focused in brain tissue from all but one of the surgeries (case 275) implying that there was an antigen driven immune response in the epileptogenic area of the brain that was removed. We also selected a sample of brain and blood from surgery case 460, which had a much lower inflammatory signature, but came from a patient who was operated on again 11 months later (surgery case 524). The T cell gene expression signature was higher in resected brain tissue from the second surgery, and the clonality score from the second surgery was double that of the first surgery indicating that T cells in the affected brain from the second surgery were more clonally focused. One public Vβ clonotype was highly significantly increased in brain tissue from the second surgery, and may explain in part the increased clonality index.

We previously found that some of the most abundant Vβ clonotypes in brain tissue from 14 RE patients were public (31). In the present study a public Vβ clonotype was the most abundant T cell clone in ten of the cases. Moreover, the Vβ CDR3 sequence of the most abundant public Vβ clonotypes were strikingly similar to Vβ CDR3 sequences that have been shown experimentally to recognize viral epitopes. We determined that two of the patients, TSC patient 595 and RE patient 597 carry HLA class I alleles that might present HCMV antigens. The Vβ CDR3 sequences in the abundant T cell clonotypes in the brain and blood of these two patients are very similar, and in one case identical, to ones that bind HCMV peptides, suggesting that the T cell clones may have arisen due to exposure to HCMV. We have no information whether the patients are seropositive for HCMV, although individuals with latent HCMV may not necessarily have detectable antibodies (32).

Five months after TSC patient 595 underwent resective surgery we found that the frequency about a third of the top 50 brain Vβ clonotypes had decreased in the blood compared with the blood collected the time of the surgery. This suggests that at least some of the T cell clones found in the brain had contracted in the periphery after removal of the brain area associated with seizure activity and inflammation. However, other public Vβ clonotypes had expanded along with an increase in circulating transitional memory effector CD8 T cells, suggesting that the expanded Vβ clonotypes could be antigen experienced CD8 T cells. The same CD8 T cell subtype was also found in the blood and resected brain tissue from Patient 597, an RE case. Based on the clinical notes, Patient 595 was seizure free and there was no report of an infection at the time the post-surgery blood was drawn. We speculate that a subclinical viral infection or an asymptomatic reactivation of a latent virus possibly HCMV may have occurred, since the Vβ CDR3 sequences of the public clones were very similar to ones that recognize an immunodominant HCMV epitope. In our analysis of future cases, we plan to use epitope specific pentamers to directly determine whether T cells isolated from resected brain tissue recognize EBV, HCMV, or influenza A antigens, and to subsequently screen for the presence of viral sequences in resected brain tissue. In multiple sclerosis patients, EBV reactive T cells have been found in perivascular spaces in the brain, and in direct contact with EBV-infected B cells (33).

The two RE cases that had the highest inflammatory signatures and clonality scores appear to represent divergent cases of T cell involvement in the disease in as much as in Patient 500 a private possibly autoreactive T cell clone comprised ~10 percent of the brain repertoire whereas in Patient 597 a public clone possibly reactive to HCMV comprised ~24 percent of the T cells. Herpesviruses have long been considered a trigger for RE and the presence of EBV and HCMV in RE brain tissue has been reported (34–38). Further it was reported that ganciclovir treatment of a patient diagnosed with early stage RE led to a cessation of seizures, directly implicating a herpesvirus in disease pathogenesis (39). HCMV and EBV are very prevalent pathogenic viruses, although children who are exposed to HCMV and EBV generally do not experience any symptoms (40, 41). Both viruses persist in a latent state in monocytes and memory B cells respectively (42, 43), and it has been shown that transition of monocytes to macrophages leads to reactivation of latent HCMV (42). Therefore, it is conceivable that HCMV could be carried into the brain by monocytes responding to seizure-driven brain inflammation, leading to viral antigen re-expression, and mobilization of HCMV reactive T cells, which could in turn result in bystander activation of autoreactive T cells. Activated T cells could permanently alter brain circuitry by directly or indirectly damaging neurons (44–47). Combining single cell RNA expression with TCR sequencing allowed us to definitively show that the dominant private and public Vβ clonotypes in the brain of a TSC patient were activated CD8 T cells. Whether the private Vβ clonotype was directed against an infectious agent or constituted an autoreactive T cell clone is not known. In TSC, FCDIIA/B, and HME clonally restricted private T cells might be directed against neoantigens expressed by dysmorphic neurons and balloon cells. We found T cells in close proximity to dysmorphic cells in sections from TSC brain tissue with a high TCR clonality index.

The TSC cases in our study cohort partitioned into either a higher or lower inflammatory group. Why there appears to be a greater involvement of the immune system in the brain in some TSC cases and not in others is not clear. It would most likely depend on the HLA alleles carried by the patient, but it may also be related to the TSC mutation itself; the mTOR pathway has been clearly implicated in the regulation of immune cell function (48). A somatic mutation arising early in gestation might be found in hematopoietic stem cells resulting in over-activation of the mTOR pathway in lymphoid and myeloid lineages. The type of mutation whether in *TSC1* or *TSC2* and the degree of mTOR over-activation could impact homeostatic immune system function. High mTOR activity enhances effector T cell effector function, promotes pro-inflammatory macrophages but suppresses the development of regulatory T cells (49, 50). In an induced seizure model, infusing regulatory T cells was found to reduce seizure activity (10). It is possible that the ameliorative effects on seizures of the rapamycin-based drug, everolimus, could in part be due to its immune modulatory action (51).

In conclusion we present evidence for T cell mediated immune responses in pediatric cases of intractable epilepsy where adaptive immunity may not have been hitherto considered a component of the disorder. Recently, new definitions of epilepsy cases that involve autoimmunity have been proposed in which the distinction is drawn between acute seizures secondary to autoimmune encephalitis as in the case of antibody-mediated encephalitides, and autoimmune-associated epilepsy for example RE (4). Based on our study cohort the latter definition may also include cases of FCD, TSC, and HME, and may support the use of modulators of adaptive immunity as potential adjunctive treatments (52–56).

## Data Availability Statement

The original contributions presented in the study are publicly available. This data can be found here: Adaptive Biotechnology immuneAccess®: https://clients.adaptivebiotech.com/pub/chang-2021-fi; DOI: 10.21417/JWC2021FI NCBI Gene Expression Omnibus: GSE168741.

## Ethics Statement

The studies involving human participants were reviewed and approved by UCLA Institutional Review Board (IRB no. 18-001048). Written informed consent to participate in this study was provided by the participants’ parent/legal guardian.

## Author Contributions

JC organized and coordinated the collection of clinical specimens and data, prepared PBMCs, carried out immunocytochemistry, and assisted with manuscript preparation. SR prepared PBMCs and BILs, and assisted with specimen collection. EF-K carried out the NanoString^®^ assays. SKL, ML, SML, RL, PL, KP, HW, GM, and AF provided surgical specimens and clinical data. NS provided diagnostic imaging data, and HV provided tissue sections and pathology reports. GO designed the study, analyzed the data, and wrote the paper with input from the co-authors. All authors contributed to the article and approved the submitted version.

## Conflict of Interest

The authors declare that the research was conducted in the absence of any commercial or financial relationships that could be construed as a potential conflict of interest.
