# Supplementary material for: Clonally Focused Public and Private T Cells in Resected Brain Tissue From Surgeries to Treat Children With Intractable Seizures
Source: Front Immunol. 2021 Apr 6;12:664344. doi: 10.3389/fimmu.2021.664344 (PMC8056262; doi:10.3389/fimmu.2021.664344)
Supplement: Supplementary file 1 [file DataSheet_1.pdf]

Table S1: Pathology, age, and gender of the pediatric epilepsy surgery patients in the study.

Case ID	Pathology	Age at surgery (yrs)	Gender	Seizure duration* (yrs.)
502	FCD IIA	1.6	F	n.a.
510	FCD IIA	13	F	11
514	FCD IIID	0.6	M	0.6
521	FCD IC	3	M	2.3
528	FCD IIA/B	15	F	14.2
535	FCD IIA/B	5	F	3
540	FCD IIID	6	M	6
549	FCD IC	13	F	2
566	FCD IIA	11	F	10.75
567	FCD IIA	13	M	12.9
575	FCD IIA	0.8	M	0.6
576	FCD IIB	11	M	0.5
580	FCD IIA	1.25	F	1.15
584	FCD IC	8	F	7.75
592	FCD IC	2	M	n.a.
600	FCD IC	16	F	15
615	FCD IIA	0.4	F	0.4
620	FCD IIA	5	M	4.25
642	FCD IIIB	13	M	10
657	FCD	9	M	3
13	HME	1.1	M	1.1
485	HME	0.6	M	0.06
596	HME	0.2	F	0.01
507	PAIS	10	F	10
526	PAIS	6	M	n.a.
531	PAIS	3	F	1.5
569	PAIS	4	M	4
656	PAIS	3	M	2.7
472	RE	9	M	3
484	RE	15	M	6
497	RE	5	M	2
500	RE	3	F	n.a.
573	RE	12	F	n.a.
597	RE	3	F	n.a.
81	TSC	11	F	10.4
97	TSC	5.5	F	5.2
99	TSC	1.9	M	1.6
100	TSC	13.3	M	13.1
106	TSC	9.6	F	9.5
133	TSC	9	M	9

148	TSC	1.3	M	1.15
166	TSC	1.6	M	1.3
186	TSC	1.7	M	1.4
275	TSC	0.9	M	0.6
297	TSC	5.2	F	4
302	TSC	12.5	F	12
367	TSC	1.3	M	1.2
460/524	TSC	3/4	M	2.8/3.7
462	TSC	4	F	3.75
539	TSC	3.4	F	n.a.
590	TSC	1.25	F	0.4
595	TSC	1.3	M	1.1

*Time from seizure onset to surgery; n.a., not available; FCD, focal cortical dysplasia;
RE, Rasmussen encephalitis; PAIS, perinatal arterial ischemic stroke; TSC, tuberous sclerosis complex

Table S2: HLA Class I molecular typing.

Case ID	Allele 1	Allele 2
595	HLA-A*03	HLA-A*26
	HLA-B*38	HLA-B*50
	HLA-C*12	HLA-C*15
597	HLA-A*01	HLA-A*24
	HLA-B*35	HLA-B*07
	HLA-C*04	HLA-C*07

HLA, Human leukocyte antigen

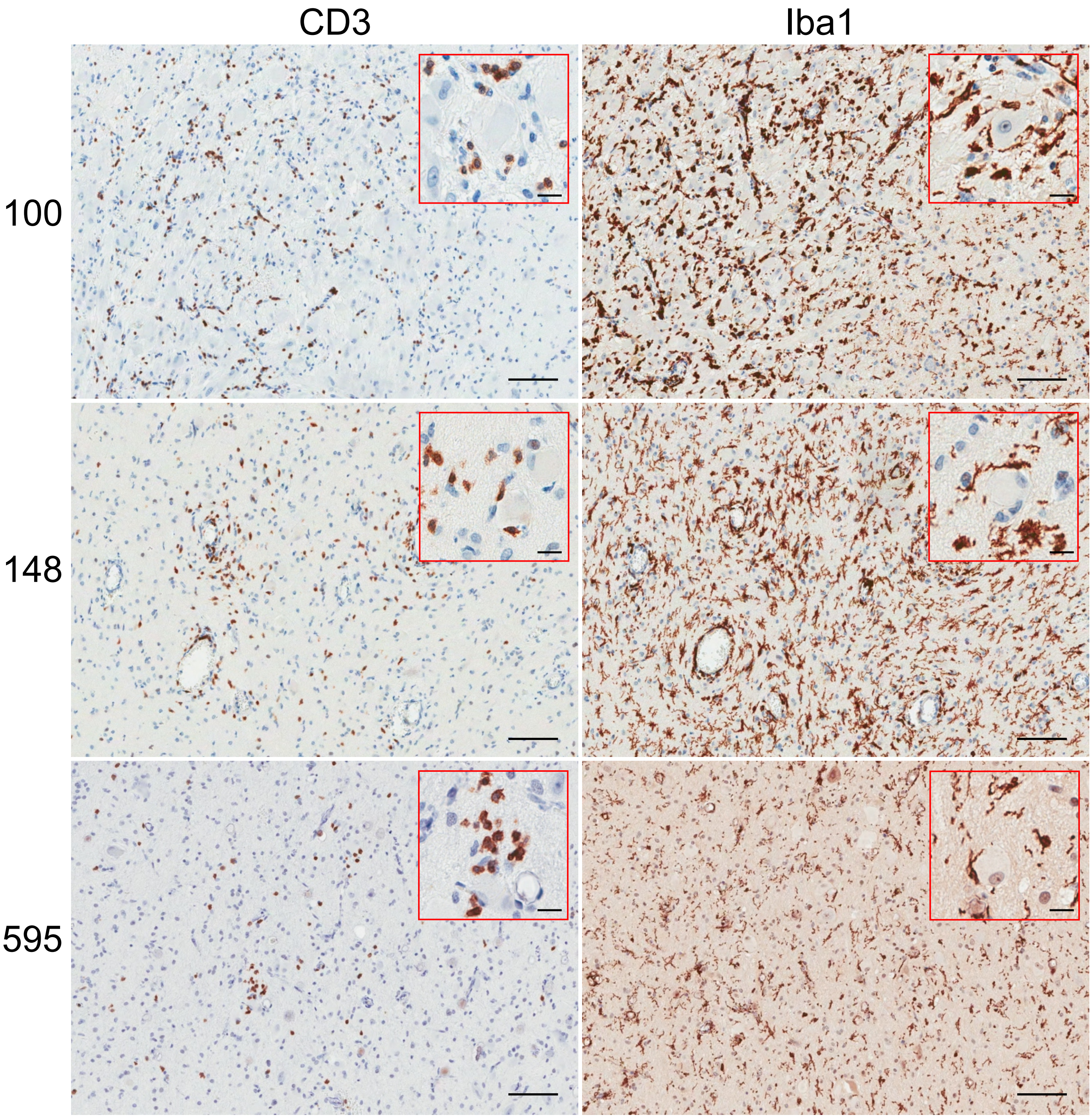

Figure S1: T cells and activated microglia in resected tubers from three TSC patients (case IDs 100, 148, and 595). CD3 and Iba1 antibody staining (red diaminobenzidine reaction product) of adjacent brains sections showing T cells and activated microglia (amoeboid-like with less ramified morphology) in close proximity to balloon cells (insets). Scale bars are 100 microns and 25 microns (insets).

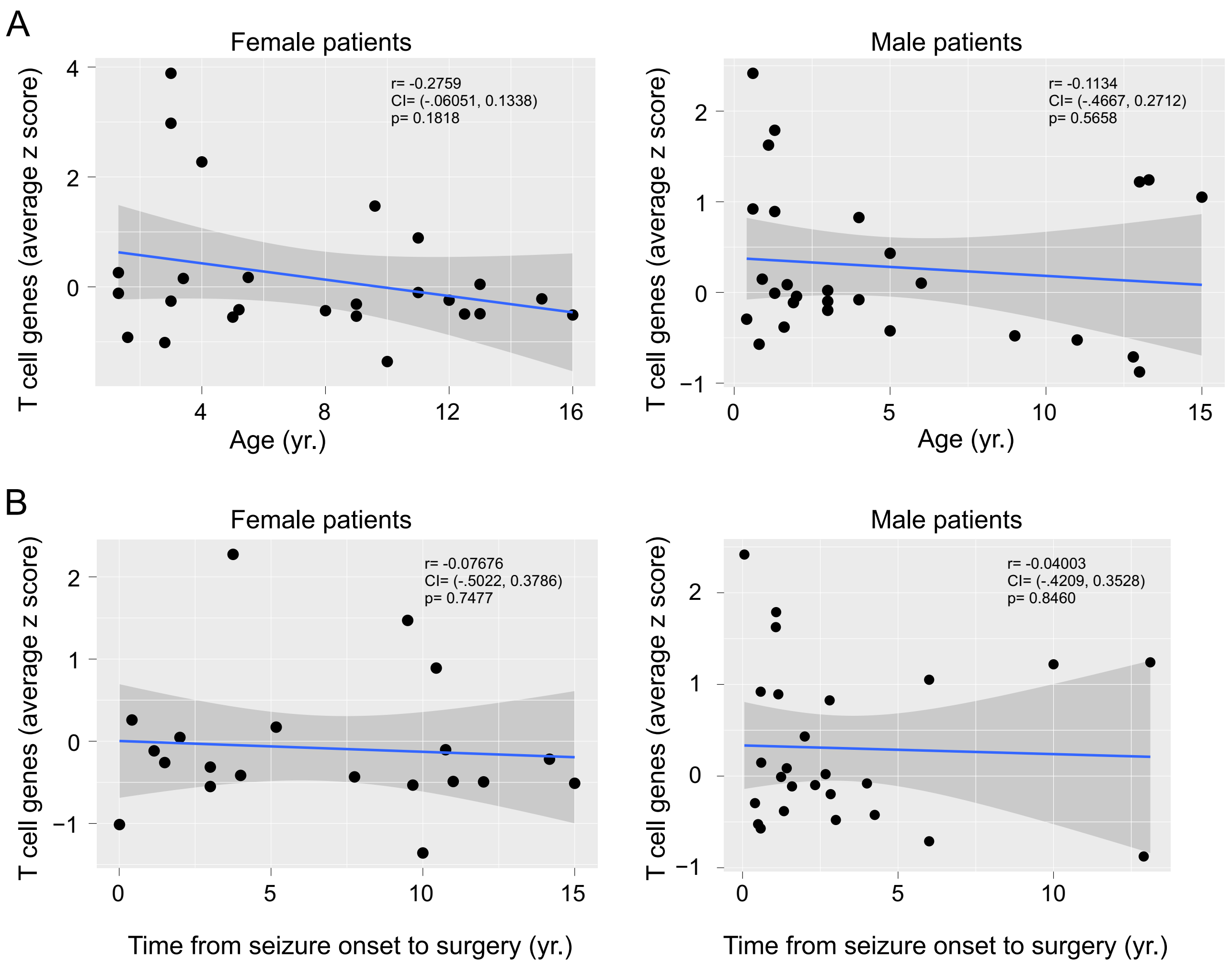

Figure S2: The extent of T cell involvement in the brain of pediatric epilepsy surgery cases is not correlated with age at surgery or seizure duration. (A) The average Z score for the T cell specific genes expressed in resected brain tissue from each surgery case (Figure 1A) was plotted against the age of the patient at the time of the surgery. (B) The average Z score for the T cell specific genes was plotted against the time from seizure onset to surgery for the patients in the study group.

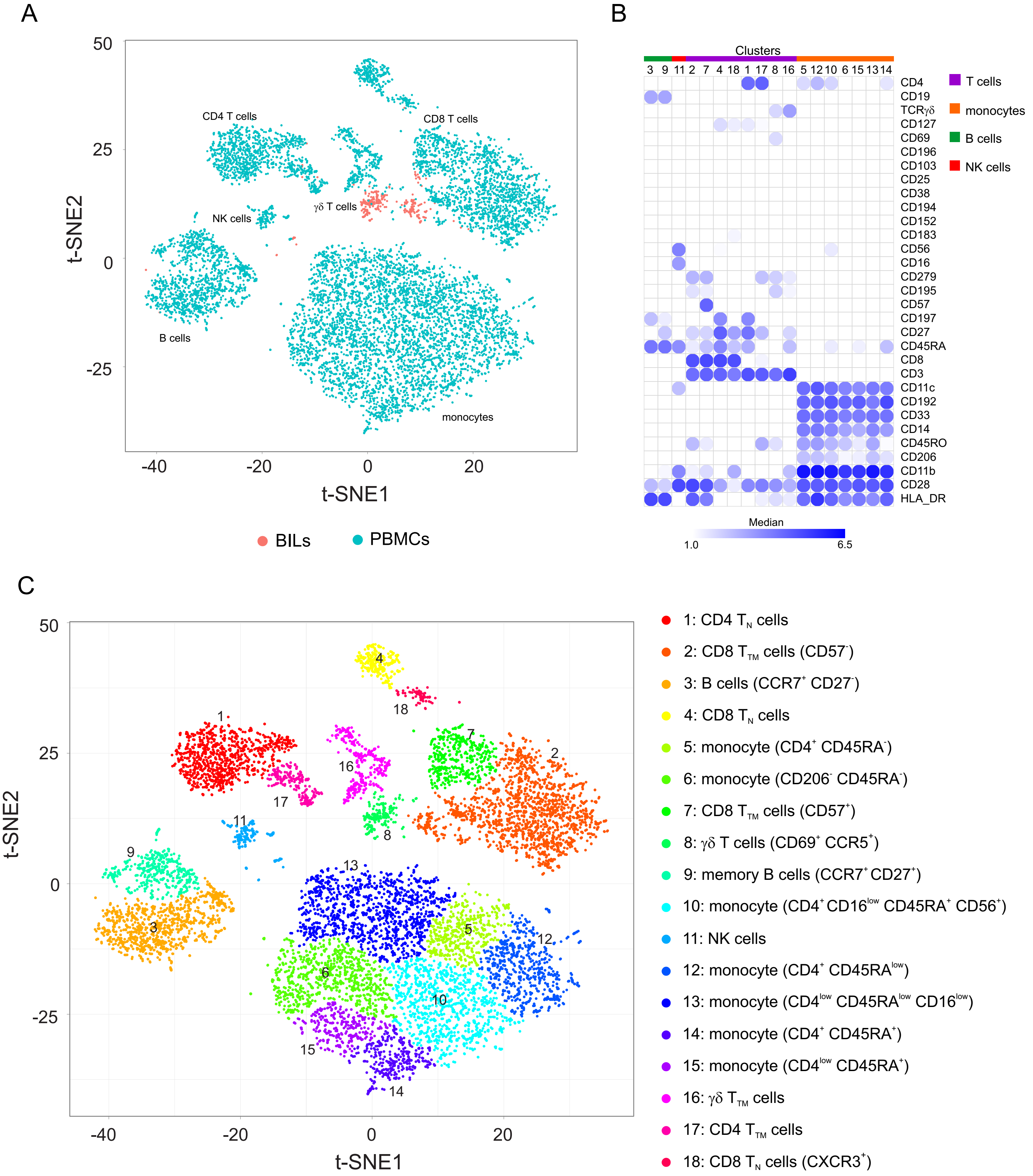
A
t-SNE2
t-SNE1
CD4 T cells
CD8 T cells
NK cells
γδ T cells
B cells
monocytes
BILs
PBMCs
B
Clusters
3 9 11 2 7 4 18 1 17 8 16 5 12 10 6 15 13 14
CD4
CD19
TCRγδ
CD127
CD69
CD196
CD103
CD25
CD38
CD194
CD152
CD183
CD56
CD16
CD279
CD195
CD57
CD197
CD27
CD45RA
CD8
CD3
CD11c
CD192
CD33
CD14
CD45RO
CD206
CD11b
CD28
HLA_DR
T cells
monocytes
B cells
NK cells
Median
1.0
6.5
C
t-SNE2
t-SNE1
1: CD4 T_N cells
2: CD8 T_{TM} cells ($CD57^-$)
3: B cells ($CCR7^+$ $CD27^-$)
4: CD8 T_N cells
5: monocyte ($CD4^+$ $CD45RA^-$)
6: monocyte ($CD206^-$ $CD45RA^-$)
7: CD8 T_{TM} cells ($CD57^+$)
8: γδ T cells ($CD69^+$ $CCR5^+$)
9: memory B cells ($CCR7^+$ $CD27^+$)
10: monocyte ($CD4^+$ $CD16^{low}$ $CD45RA^+$ $CD56^+$)
11: NK cells
12: monocyte ($CD4^+$ $CD45RA^{low}$)
13: monocyte ($CD4^{low}$ $CD45RA^{low}$ $CD16^{low}$)
14: monocyte ($CD4^+$ $CD45RA^+$)
15: monocyte ($CD4^{low}$ $CD45RA^+$)
16: γδ T_{TM} cells
17: CD4 T_{TM} cells
18: CD8 T_N cells ($CXCR3^+$)

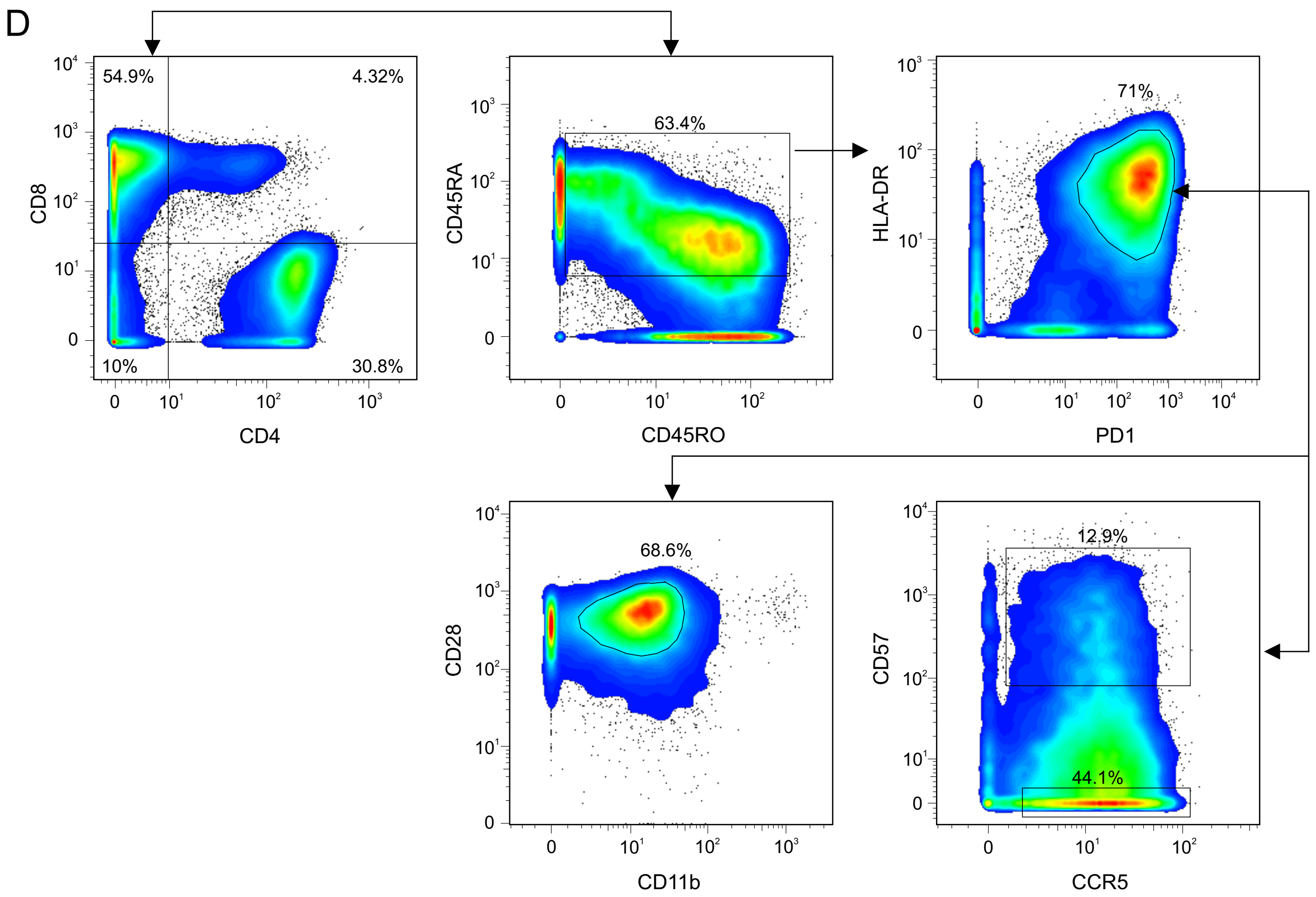

Figure S3: Immune cell profiles of peripheral blood mononuclear cells and brain-infiltrating leukocytes from Patient 597 diagnosed with Rasmussen encephalitis. (A) The expression of 31 immune cell markers on each $CD45^+$ cell was reduced to coordinates in two-dimensional space using the t-SNE algorithm. The resulting t-SNE plot shows that limited number of brain-infiltrating leukocytes (BILs; orange dots corresponding to individual cells) isolated from resected brain tissue are predominantly CD8 T cells and γδ T cells. Turquoise dots correspond to individual peripheral blood mononuclear cells (PBMCs) from the same patient. Ten thousand $CD45^+$ PBMCs were selected at random for the calculation. (B and C) The 2D coordinates were used to identify the types of $CD45^+$ immune cells in the BILs and PBMCs by implementing a density dependent clustering algorithm (ClusterX), which partitioned the t-SNE plot into 18 clusters. Immune cell phenotypes were assigned to each cluster based on the median expression of each marker in each cluster. (D) To confirm the presence of $CD45RO^+$ $CD45RA^+$ transitional memory CD8 T cells (CD8 TTM cells) in BILs and PBMCs (Clusters 2 and 7) the original FCS file of marker expression on PBMCs was re-analyzed using FLOWJo. Manual gating of $CD45^+$ $CD3^+$ $CD8^+$ confirmed the presence of $CD57^+$ and $CD57^-$ CD45RO and CD45RA double positive T cells.

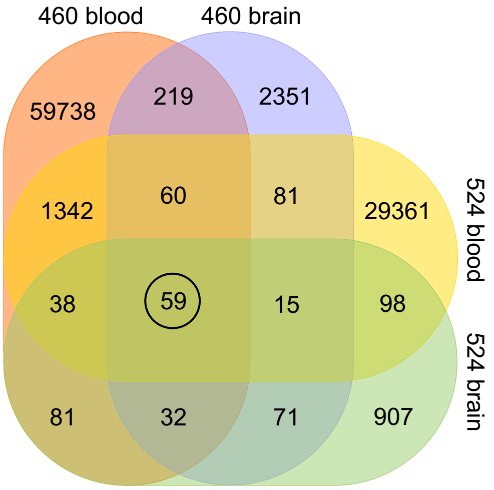

Figure S4: Changes in the TCR repertoire between the first (case 460) and second surgery (case 524). One of the TSC patients in the study cohort was operated on twice 11 months apart (surgery cases 460 and 524). The venn diagram shows the extent of overlap between the sampled T cell repertoires (unique Vβ CDR3 amino acid sequences) in specimens of blood and resected brain tissue from both surgeries. The 59 overlapping Vβ CDR3 amino acid sequences comprised 7.37%, 9.56% , 6.76%, and 19.22% of the sampled 460 blood, 460 brain, 524 blood and 524 brain repertoires respectively.

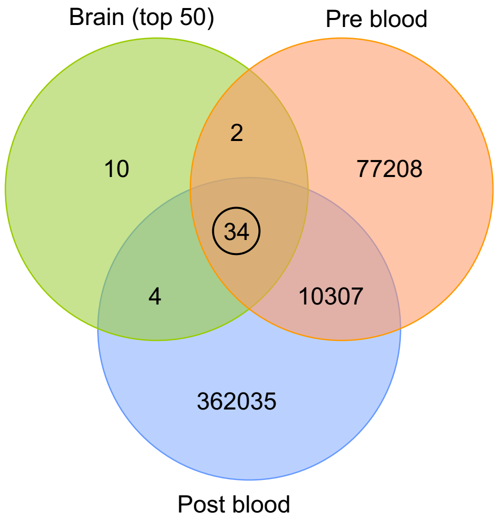

Figure S5: Overlap between the top 50 brain TCR clonotypes in blood before and after surgery. Blood was collected from TSC patient 595 five months after surgery. The venn diagram shows the extent of overlap between the sampled T cell repertoires (unique Vβ CDR3 amino acid sequences) in specimens of blood obtained at the time of the surgery (Pre blood), and 5 months after the surgery (Post blood), and the top 50 T cell clonotypes found in resected brain tissue. The 34 overlapping Vβ CDR3 amino acid sequences comprised 15.36%, 0.26%, and 0.12% of the top brain, pre blood and post blood sampled repertoires respectively.

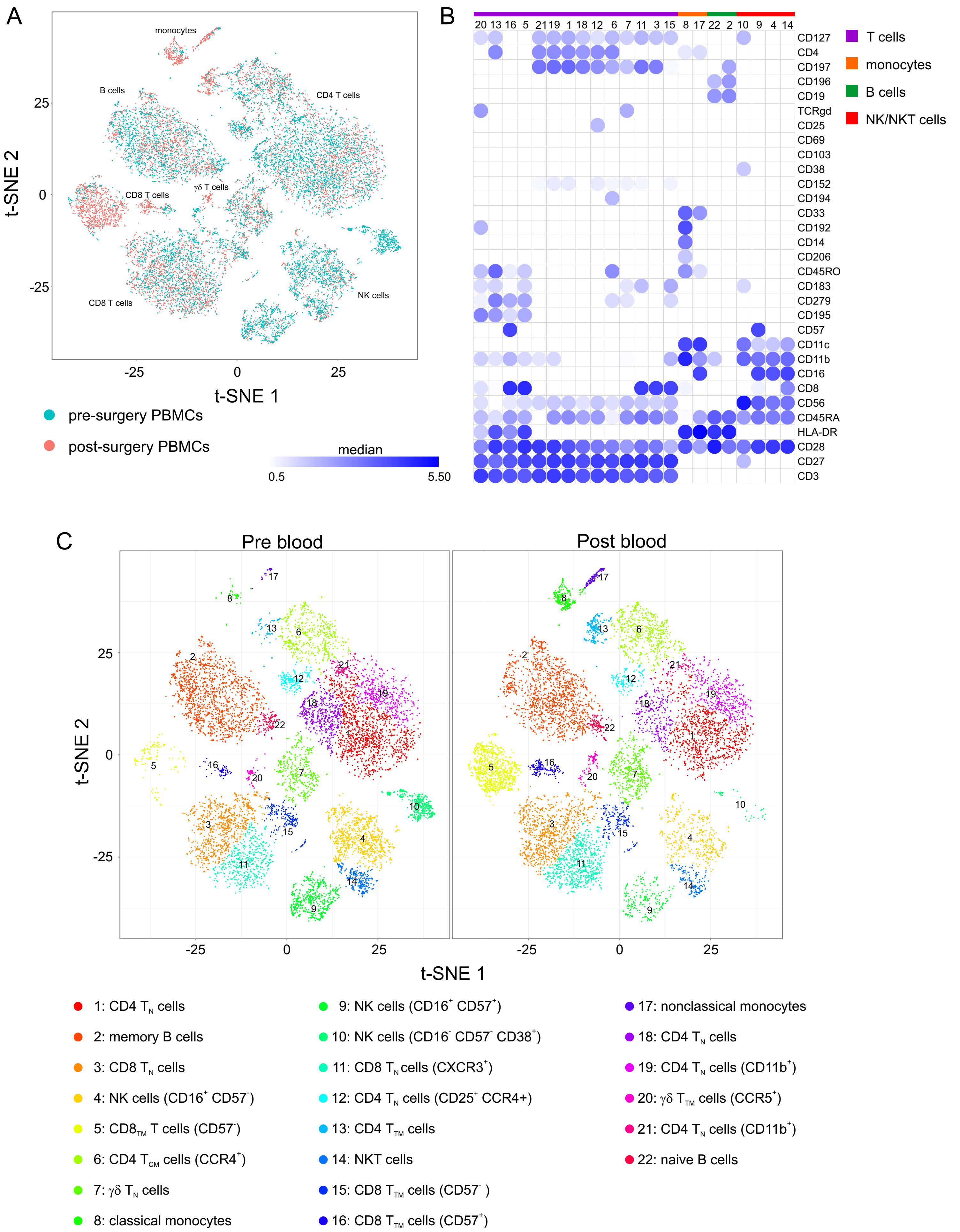
A
monocytes
B cells
CD4 T cells
CD8 T cells
γδ T cells
NK cells
t-SNE 1
t-SNE 2
pre-surgery PBMCs
post-surgery PBMCs
B
20 13 16 5 21 19 1 18 12 6 7 11 3 15 8 17 22 2 10 9 4 14
CD127
CD4
CD197
CD196
CD19
TCRgd
CD25
CD69
CD103
CD38
CD152
CD194
CD33
CD192
CD14
CD206
CD45RO
CD183
CD279
CD195
CD57
CD11c
CD11b
CD16
CD8
CD56
CD45RA
HLA-DR
CD28
CD27
CD3
T cells
monocytes
B cells
NK/NKT cells
median
0.5
5.50
C
Pre blood
Post blood
t-SNE 1
t-SNE 2
1: CD4 T_N cells
2: memory B cells
3: CD8 T_N cells
4: NK cells ($CD16^+$ $CD57^-$)
5: $CD8_{TM}$ T cells ($CD57^-$)
6: CD4 T_{CM} cells ($CCR4^+$)
7: γδ T_N cells
8: classical monocytes
9: NK cells ($CD16^+$ $CD57^+$)
10: NK cells ($CD16^-$ $CD57^-$ $CD38^+$)
11: CD8 T_N cells ($CXCR3^+$)
12: CD4 T_N cells ($CD25^+$ CCR4+)
13: CD4 T_{TM} cells
14: NKT cells
15: CD8 T_{TM} cells ($CD57^-$)
16: CD8 T_{TM} cells ($CD57^+$)
17: nonclassical monocytes
18: CD4 T_N cells
19: CD4 T_N cells ($CD11b^+$)
20: γδ T_{TM} cells ($CCR5^+$)
21: CD4 T_N cells ($CD11b^+$)
22: naive B cells

D

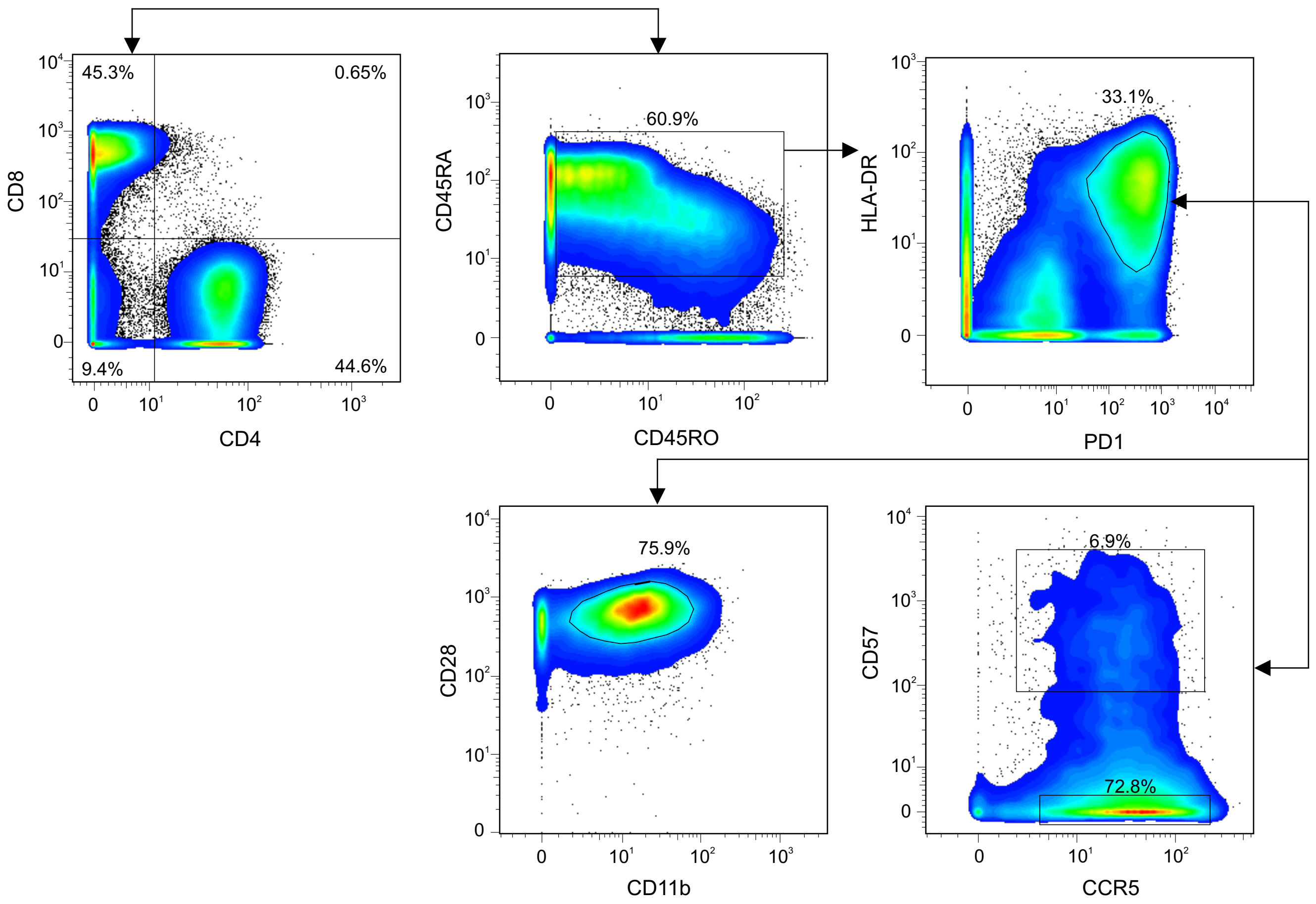

Figure S6: Immune cell profiles of peripheral blood mononuclear cells from Patient 595, diagnosed with Tuberous sclerosis complex, pre-and post-surgery. (A) The expression of 31 immune cell markers on each CD45$^+$ cell was reduced to coordinates in two-dimensional space using the t-SNE algorithm. The resulting t-SNE plot shows immune cells (pre-surgery- turquoise dots corresponding to individual cells; post-surgery- orange dots). Ten thousand CD45$^+$ PBMCs were selected at random for the calculation. (B and C) The 2D coordinates were used to identify the types of CD45$^+$ immune cells in the pre- and post-surgery PBMCs by implementing a density dependent clustering algorithm (ClusterX), which partitioned the t-SNE plot into 22 clusters. Immune cell phenotypes were assigned to each cluster based on the median expression of each marker in each cluster. (D) To confirm the presence of CD45RO$^+$ CD45RA$^+$ transitional memory CD8 T cells (CD8 T_{TM} cells) in pre- and post-surgery PBMCs (Clusters 15 and 16) the original FCS file of marker expression on post-surgery PBMCs was re-analyzed using FLOWJo. Manual gating of CD45$^+$ CD3$^+$ CD8$^+$ confirmed the presence of CD57$^+$ and CD57$^-$ CD45RO and CD45RA double positive T cells.

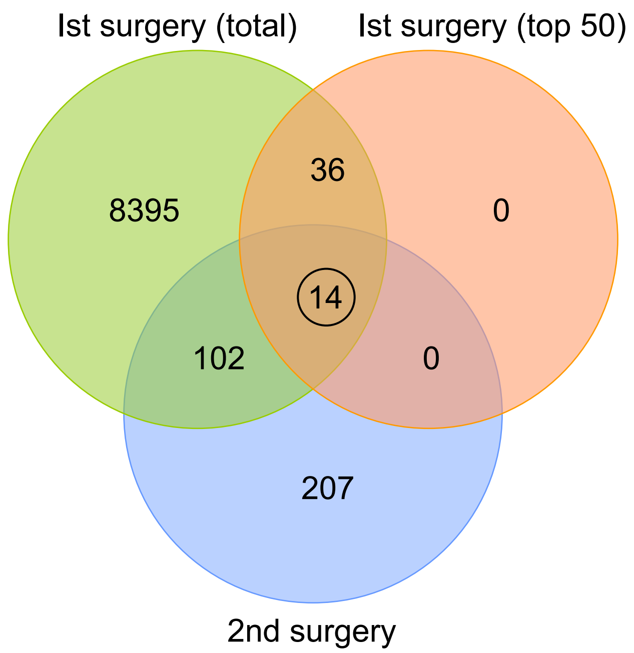

Figure S7: Overlap between TCR clonotypes in brain specimens from the first and second surgeries. TSC patient 595 was operated on twice 11 months apart. The venn diagram shows the extent of overlap between T cell clonotypes (unique Vβ CDR3 amino acid sequences) in brain-infiltrating leukocytes from the second surgery and T cell clonotypes in resected brain tissue from the first surgery (top 50 and total clonotypes). The 14 overlapping Vβ CDR3 amino acid sequences comprised 5.89% of the top 50 Vβ sequences from the first surgery and 8.44% of the T cells from the second surgery.
